# Circuit-guided population acclimation of a synthetic microbial consortium for improved biochemical production

**DOI:** 10.1038/s41467-022-34190-z

**Published:** 2022-11-07

**Authors:** Chae Won Kang, Hyun Gyu Lim, Jaehyuk Won, Sanghak Cha, Giyoung Shin, Jae-Seong Yang, Jaeyoung Sung, Gyoo Yeol Jung

**Affiliations:** 1 Department of Chemical Engineering, Pohang University of Science and Technology, 77 Cheongam-Ro, Nam-Gu, Pohang, Gyeongbuk 37673 Korea; 2 Creative Research Initiative Center for Chemical Dynamics in Living Cells, Chung-Ang University, 84 Heukseok-Ro, Dongjak-gu, Seoul 06974 Republic of Korea; 3 Department of Chemistry, Chung-Ang University, 84 Heukseok-Ro, Dongjak-gu, Seoul 06974 Republic of Korea; 4 School of Interdisciplinary Bioscience and Bioengineering, Pohang University of Science and Technology, 77 Cheongam-Ro, Nam-Gu, Pohang, Gyeongbuk 37673 Korea; 5 Centre for Research in Agricultural Genomics (CRAG), CSIC-IRTA-UAB-UB, Campus UAB, Bellaterra, Barcelona, 08193 Spain

**Keywords:** Metabolic engineering, Synthetic biology

## Abstract

Microbial consortia have been considered potential platforms for bioprocessing applications. However, the complexity in process control owing to the use of multiple strains necessitates the use of an efficient population control strategy. Herein, we report circuit-guided synthetic acclimation as a strategy to improve biochemical production by a microbial consortium. We designed a consortium comprising alginate-utilizing *Vibrio* sp. dhg and 3-hydroxypropionic acid (3-HP)-producing *Escherichia coli* strains for the direct conversion of alginate to 3-HP. We introduced a genetic circuit, named “Population guider”, in the *E. coli* strain, which degrades ampicillin only when 3-HP is produced. In the presence of ampicillin as a selection pressure, the consortium was successfully acclimated for increased 3-HP production by 4.3-fold compared to that by a simple co-culturing consortium during a 48-h fermentation. We believe this concept is a useful strategy for the development of robust consortium-based bioprocesses.

## Abstract (web summary)

Achieving stable production by co-culturing multiple microorganisms is often challenging due to difficulties in controlling its population. Here, the authors develop a “population guider” that can acclimate a population to higher production.

## Introduction

Microorganisms grow as consortia across domains by optimizing community structures for their survival by acclimatizing to their environments^1–4^. They communicate by the secretion and uptake of metabolites or chemicals typically used for cellular communication^5^. These communications facilitate diverse social interactions (e.g., commensalism, cooperation, amensalism, and competition). One of the important features that is observed in these community structures is the division of labor^6–8^. While community participants usually compete for limited resources, they often complement each other by performing different tasks, and also exhibit emergent properties, which enables the expression of improved phenotypes (i.e., fitness) compared to those expressed in independent cultures^2,9–11^.

Based on these facts, microbial consortia have been studied as attractive bioprocessing platforms for biochemical production^7,12–15^. While single-cell engineering of genetically well-known microorganisms has been performed widely, it is difficult to efficiently introduce large or complicated metabolic pathways, which are required for the utilization of non-conventional substrates (e.g., single-carbon gases, alginate) or production of complex chemicals (e.g., natural products). Microorganisms that naturally exhibit such abilities can be considered potential hosts; however, their use in bioprocessing is often limited owing to insufficient engineering tools or understanding^16^. On the contrary, the use of a microbial consortium does not require extensive engineering and can exploit the innate abilities of microorganisms that have been optimized through evolution. Furthermore, the division of labor achieved by the spatial compartmentalization of metabolic pathways in different cells may relieve the metabolic burden^13,17^ and provide additional control for balancing various activities in the pathway^18–20^. Indeed, in the past years, several synthetic consortia have been developed for the conversion of various substrates (e.g., cellobiose^21^, cellulose^22^, carbon monoxide^23^) and production of biochemicals (methyl halides^24^, oxygenated taxanes^19^, muconic acid^25^, vitamin C^26^, hydroxytyrosol^27^, cadaverine^28^).

While the utilization of microbial consortia is promising, a critical challenge is the control of process performance for the maximization of production efficiencies, which is primarily governed by the population composition^29^. Furthermore, there were also efforts in developing artificial quorum sensing circuits^21,30–32^ for programmed growth control of consortium participants depending on population densities. In addition, to achieve stability in co-cultures, symbiotic communities were designed mainly via creating mutualistic interactions, including carbon cross-feeding^33^ and feeding a growth-inhibiting byproduct to another member as a sole carbon source^19,23^.

While these efforts have successfully improved biochemical production, and also highlighted the importance of population control, it remains difficult to predict and control the optimal population composition of each participant for biochemical production. In particular, the microbial population of a consortium with different physiological attributes is rarely controllable simply using initial conditions, because the growth of each cell is significantly affected by the medium composition that changes dynamically throughout the cultivation period. Therefore, the optimization process relies on pre-determined empirical parameters that can only be determined through labor-intensive experimental trials.

In this study, we report a population-guiding strategy for improving biochemical production by a microbial consortium based on its synthetic acclimation. Specifically, we designed a genetic circuit, named “population guider”, for the degradation of specific compounds inhibiting the growth of the consortium when a target chemical is produced. In the presence of the growth inhibitor, a population guider tightly associates the growth of a microbial consortium with the biochemical production capability. Therefore, a population is expected to be autonomously acclimated for improved biochemical production by its optimization even under dynamically changing environments. As a model system, we applied this strategy to a consortium of *Vibrio* sp. dhg^34,35^ and an engineered *E. coli* strain for the direct production of 3-hydroxypropionic acid (3-HP), an important platform chemical^36^, from alginate, a promising non-conventional feedstock material obtainable from marine biomass. We chose ampicillin as a growth-inhibiting compound and developed a population guider to express the ampicillin degradation gene (*bla*) in response to the 3-HP concentration. When *Vibrio* sp. dhg was co-cultivated with a 3-HP-producing *E. coli* harboring the population guider in the presence of ampicillin, notably, it was observed that 3-HP production was drastically increased (up to 4.3-fold) with the minimized accumulation of acetate, a key intermediate in co-cultures. A population analysis revealed that the improvement occurred due to changes in the population compositions of co-cultures based on the concentrations of the growth-inhibiting compounds. Collectively, these results suggest that the concept of synthetic acclimation can serve as an efficient strategy for improving the production of diverse value-added biochemicals by microbial consortia.

## Results

### Microbial consortium for production of 3-HP from alginate

**Fig. 1:**
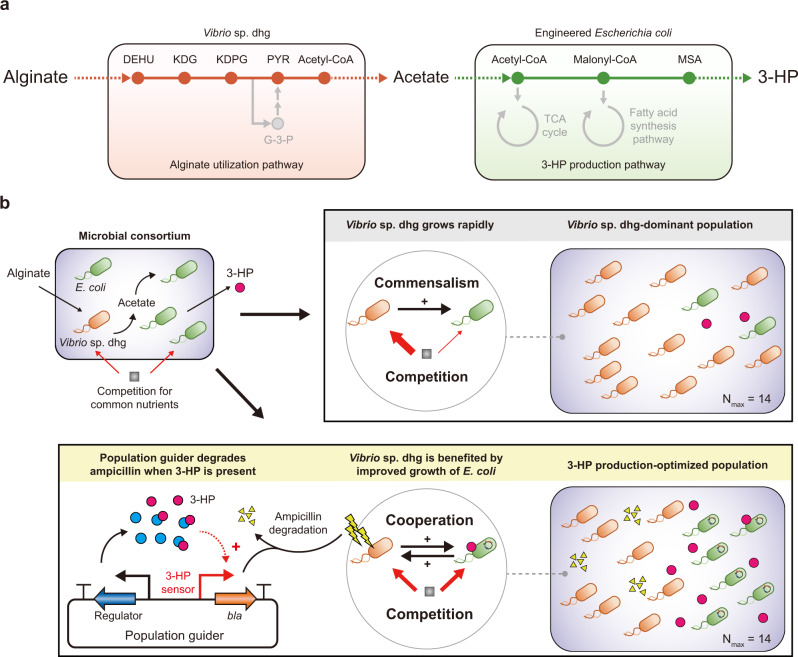
Overall strategy of genetic circuit-guided acclimation of the microbial consortium for improved 3-hydroxypropionic acid (3-HP) production. **a** Schematic diagram of the 3-HP biosynthesis from alginate by the microbial consortium comprising *Vibrio* sp. dhg and *E. coli. Vibrio* sp. dhg assimilates alginate and secretes acetate, whereas *E. coli* produces 3-HP by re-utilizing the secreted acetate. Abbreviations: 3-HP, 3-hyroxypropionic acid; DEHU, 4-deoxy-L-erythro-5-hexoseulose uronate; KDG, 2-keto-3-deoxygluconate; KDPG, 2-keto-3-deoxy-6-phosphogluconate; PYR, pyruvate; G-3-P, glyceraldehyde 3-phosphate; MSA, malonate semialdehyde. **b** A comparison of the microbial consortium-based strategy with genetic circuit-based population guiding. Without population control, the population is dominated by *Vibrio* sp. dhg, resulting in the wasteful utilization of resources and a low production of 3-HP. The introduction of the population guider into *E. coli* leads to the establishment of a cooperative interaction between the two microorganisms and allows the consortium to optimize the population for improving 3-HP production by synthetic acclimation.

A synthetic microbial consortium was constructed for the production of 3-HP from alginate, in which an alginate-utilizing *Vibrio* sp. dhg strain and a 3-HP producing *E. coli* strain were co-cultured (Fig. 1a). *Vibrio* sp. dhg has a 42-kb cluster that encodes proteins that metabolize alginate^34^ and release acetate as a major byproduct. While *E. coli* cannot utilize alginate, its biochemical production from various carbon sources, including acetate, has been performed frequently^37–40^. In the current study, *E. coli* was engineered to produce 3-HP from acetate (Supplementary Note 1); the resulting ECFHPS strain produced 272.82 mg/L of 3-HP from 10 g/L of acetate (Supplementary Fig. 1 and Supplementary Data 1). Therefore, it was expected that co-culturing could be effectively used to convert alginate into 3-HP with acetate as an intermediate.

To facilitate efficient 3-HP production from alginate, we devised a genetic circuit-based population-guiding strategy (Fig. 1b). Given that only *Vibrio* sp. dhg serves as a beneficial strain (i.e., by providing acetate) for *E. coli* by metabolizing alginate, whereas *E. coli* does not significantly promote the growth of *Vibrio* sp. dhg, the two strains were expected to exhibit a commensal relationship, which would lead to the dominance of *Vibrio* sp. dhg in co-cultures. Consequently, the excess growth of *Vibrio* sp. dhg would inevitably reduce the *E. coli* population, as the former would compete for other nutrients in the medium^41^. To obtain the optimal population ratio for 3-HP production, we established a cooperative relationship between the two strains by introducing a population guider in *E. coli*. This population guider was designed to express the *bla* gene for the degradation of ampicillin in the presence of 3-HP in a medium. In the presence of ampicillin, which inhibits the growth of the microbial strains (primarily *Vibrio* sp. dhg), *E. coli* served as a beneficial strain by improving the growth fitness of *Vibrio* sp. dhg. We expected that strains would be acclimated to the increased production of 3-HP by cooperative survival under selection pressure.

### Cultivation of consortium without population control

**Fig. 2:**
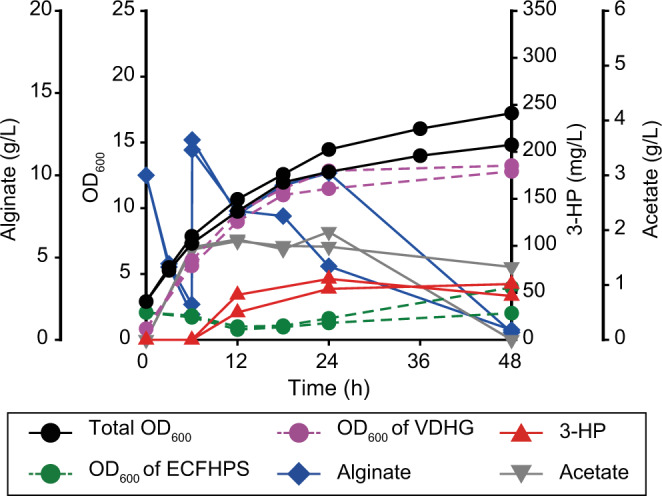
3-HP production from alginate by the microbial consortium comprising VDHG and ECFHPS strains. Fermentation profile of the co-culture of the VDHG and ECFHPS strains. The left *y*-axis and left *y*-offset represent OD_600_ (black circles) and consumed alginate (blue diamond, g/L), respectively. Estimated OD_600_ of the VDHG (purple circles) and ECFHPS (green circles) strains was also provided. The right *y*-axis and right *y*-offset represent 3-HP (red triangles, mg/L) and acetate (grey inverted triangles, g/L) levels, respectively. Source data are provided as a Source Data file.

Initially, we constructed a synthetic consortium by co-culturing *Vibrio* sp. dhg and *E. coli* and examined whether 3-HP could be produced directly from alginate. To monitor the population composition, we labeled *Vibrio* sp. dhg and ECFHP (3-HP-producing *E. coli* W, Supplementary Data 1) strains by introducing gene expression cassettes for the constitutive expression of *mcherry* and *sgfp* (Supplementary Fig. 2), which led to the formation of the VDHG and ECFHPS strains, respectively (Supplementary Data 1). To initiate the culture, cells of the VDHG and ECFHPS strains (with the cell cultures having concentrations corresponding to OD_600_ values 0.5 and 2, respectively) were used as inoculum, considering that *Vibrio* sp. dhg cultured in the presence of alginate exhibited a considerably higher growth rate (0.83 h^−1^) than that of *E. coli* cultured in the presence of acetate (0.16 h^−1^) (Supplementary Fig. 3). After the culture was initiated in a minimal medium supplemented with alginate, an immediate increase in the OD_600_ value was observed (Fig. 2a). Along with cell growth, acetate accumulation was also observed in the medium (1.67 g/L), indicating that *Vibrio* sp. dhg responded positively to the co-culture conditions (in terms of growth). At 12 h, 3-HP was also detected (38.45 mg/L), which confirmed that 3-HP can be produced directly from alginate using the consortium approach. Nevertheless, only a small quantity of 3-HP (52.95 mg/L from 20 g/L of alginate) was produced by the consortium even when the acetate level was lowered. The significant accumulation of acetate (1.83 g/L at maximum) indicated the low efficiency of 3-HP production (i.e., insufficient activity of the 3-HP-producing *E. coli*). The population composition analysis revealed that the population ratio of ECFHPS reduced significantly with time and remained low (lowest: 8.25% at 18 h, Fig. 2b). These results indicate that the overgrowth of *Vibrio* sp. dhg should be avoided for efficient 3-HP production.

To investigate the effect of population ratios to 3-HP production, we tested four different inoculum sizes of the VDHG and ECFHPS strains (0.5:4, 0.5:1, 0.1:2.4, and 2.4:0.1), which were varied from the 0.5:2 ratio (Supplementary Fig. 4). As a result, the changes of the population ratio affected the 3-HP production significantly; the 3-HP titers at 48 h varied from almost zero to 211.05 mg/L (a 4.0-fold increase), implying the importance of a consortium population to 3-HP production. In particular, when the initial amount of VDHG is high, 3-HP production greatly decreased likely due to poor growth of the ECFHPS strain. Despite the inoculum size control, acetate was still consistently observed at high levels, implying significant room for further optimization. It was also found that generated 3-HP was also often decreased likely due to its degradation by either of the two microorganisms after alginate was depleted, indicating the necessity of efficient carbon utilization; the degradation of 3-HP was observed in another bacterium previously^42^. With the confirmed importance of the population ratio control, we applied the population control strategy to optimize production by this consortium.

### Population guider for 3-HP-dependent growth regulation

To guide the microbial population for improved 3-HP production, we constructed a population guider and tested its controllability in terms of the growth of the consortium depending on the presence of 3-HP. We utilized a 3-HP-responsive transcription factor (*C4-lysR*) and its cognate promoter (P_C4M_) from a recently characterized 3-HP inducible system in *Pseudomonas denitrificans*^43–45^ (Fig. 1b). For the effective control of 3-HP-dependent *bla* expression, we assessed two 5’-UTR sequences (weak and strong) with different predicted expression levels (Supplementary Table 1). The ECPG1 strain harboring the pPopG1 plasmid with the weak 5’-UTR for *bla* was unable to grow in the absence of 3-HP (Supplementary Fig. 5a). Conversely, when the strong 5’-UTR was used (in the ECPG2 strain), the strain could grow regardless of the presence of 3-HP, probably owing to the high basal expression of *bla* (Supplementary Fig. 5b). Therefore, the population guider with the weak 5’-UTR for *bla* expression was selected for subsequent studies.

**Fig. 3:**
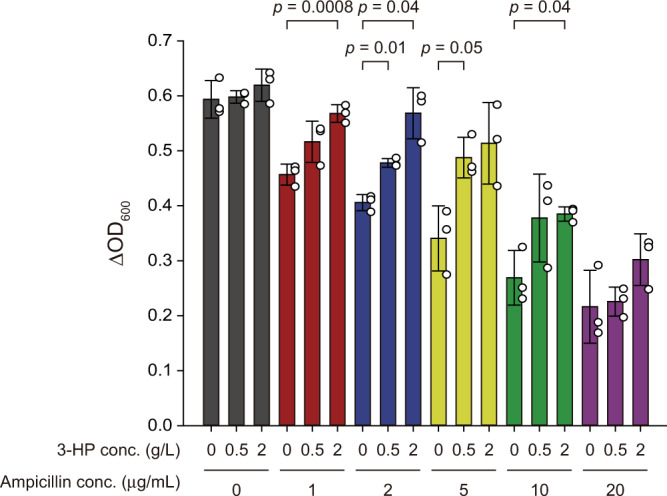
3-HP dependent growth control of the microbial consortium in presence of ampicillin. Comparison of biomass formation (ΔOD_600_, the difference in OD_600_ values at 0 h and 24 h) in various combinations of 3-HP (0, 0.5, and 2 g/L) and ampicillin (0, 1, 2, 5, 10, and 20 µg/mL) concentrations. Error bars represent the standard deviations of triplicate biological experiments (*n* = 3) and their center indicates a mean value. *P* values were calculated using Student’s t-test (two-tailed). White dot indicates actual data point. Source data are provided as a Source Data file.

Next, we assessed whether the growth of the consortium could be controlled by the exogenous addition of 3-HP using the developed population guider (Fig. 3). We prepared an inoculum by mixing the VDHG and ECPG (*E. coli* W harboring the pPopG plasmid, Supplementary Data 1) strains at a ratio of 1:4, considering their growth rates (see above). We monitored the growth of the consortium in the presence of sub-lethal concentrations of ampicillin (0, 1, 2, 5, 10, and 20 µg/mL, Supplementary Fig. 6). For the inducible expression of the *bla* gene, three different concentrations of 3-HP (0, 0.5, and 2 g/L) were used. Notably, after 24 h of cultivation, successful growth control was observed; the consortium generally exhibited better growth in the presence of 3-HP when the ampicillin concentrations ranged from 1 to 10 µg/mL. The differences in biomass formation depending on the presence of 3-HP were generally more pronounced when the concentration of ampicillin was higher. However, when the ampicillin concentration was excessively high (20 µg/mL), the growth benefit was unclear, probably owing to the excessive inhibition of the growth of both strains. Nevertheless, this result indicates the potential of the population guider for regulating the growth of the microbial consortium in response to the presence of 3-HP in the medium.

### Acclimation of a microbial population for 3-HP production

**Fig. 4:**
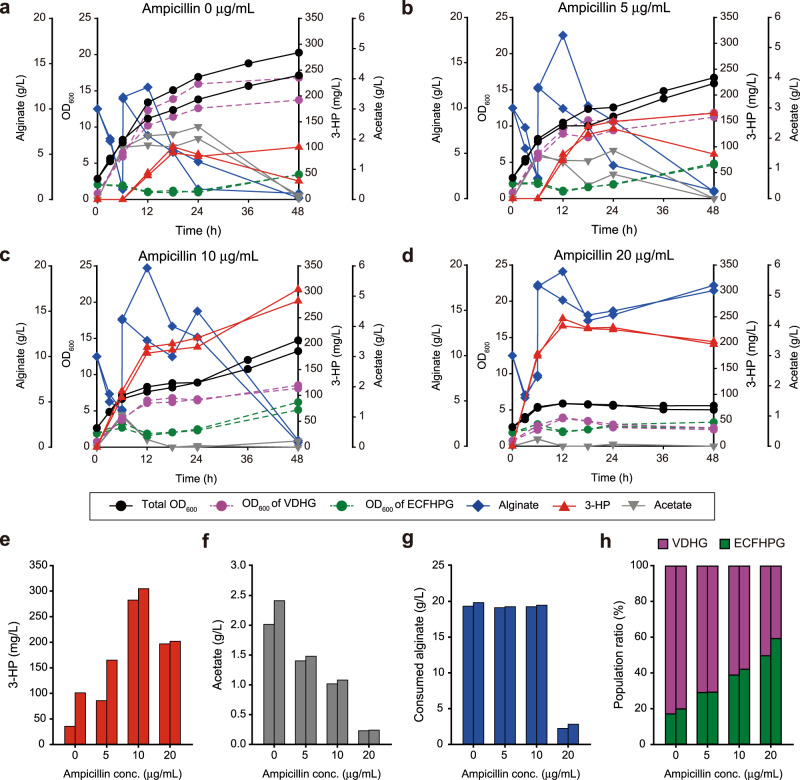
Improved 3-HP production upon the synthetic acclimation of the microbial consortium. **a**–**d** Fermentation profiles of the co-culture of the VDHG and ECFHPG strains with the addition of (**a**) 0, (**b**) 5, (**c**) 10, and (**d**) 20 µg/mL ampicillin. The left *y*-axis represents OD_600_ of cultures (black circles). Estimated OD_600_ of the VDHG (purple circles) and ECFHPG (green circles) strains was also provided. The left *y*-offset, right *y*-axis and right *y*-offset represent alginate (blue diamonds, g/L), 3-HP (red triangles, mg/L) and acetate (grey inverted triangles, g/L) levels, respectively. **e–h** Comparison of (**e**) 3-HP levels, (**f**) acetate levels, (**g**) consumed alginate, and (**h**) population ratios depending on ampicillin concentrations (0, 5, 10, and 20 μg/mL) at 48 h. The purple and green bars indicate the population ratio of VDHG and ECFHPG strains, respectively. **a–h** Source data are provided as a Source Data file.

We applied the population-guiding strategy for the robust production of 3-HP from alginate by co-culturing the VDHG strain with the ECFHPG strain (an engineered ECF strain harboring the pPopG-3HP plasmid, Supplementary Data 1) in the presence of ampicillin (Fig. 4a–d for the culture profiles and Fig. 4e–h for their comparisons). In the initial attempt, we added 5 µg/mL of ampicillin, and the fermentation profile was compared to that observed in the absence of ampicillin (Fig. 4 and Supplementary Table 2). In the absence of ampicillin, the culture profiles and levels of 3-HP production (68.35 mg/L) were almost identical with those observed for the initial consortium, in which VDHG and ECFHPS were co-cultured (Fig. 4a). This observation indicates that the population guider did not affect the cultures. When 5 µg/mL of ampicillin was added, the growth rate decreased marginally along with the alginate consumption rate (Fig. 4b). Nevertheless, the increased 3-HP titer (125.40 mg/L) and low levels of accumulated acetate (1.44 g/L at most) were notable observations, indicating the improved 3-HP production following population control. Subsequently, we investigated a potential change in the population of the consortium and observed that the ratio of ECFHPG increased from 18.50% to 29.15% of the total population at 48 h (Fig. 4h). Such population changes resulted in the redirection of carbon flux toward 3-HP production, represented by a 1.89-fold increase in the C-mole yield of 3-HP (Supplementary Table 2).

We increased the selection pressure by adding 10 µg/mL of ampicillin, at which point the growth of the VDHG strain was almost completely inhibited (Supplementary Fig. 6). Although the addition of excess ampicillin strongly inhibited the growth of the consortium in the absence of the population regulation module (Supplementary Fig. 7), when the population guider was used, the OD_600_ value of the consortium reached 13.99 (Fig. 4c). This indicates the successful cooperative interaction between the two microorganisms. The increase in selection pressure further increased the 3-HP titer (293.55 mg/L), a 4.3-fold increase compared to that observed in the absence ampicillin (68.35 mg/L) after a 48-h fermentation period. Acetate accumulation was significantly low (1.05 g/L at 6 h) and undetectable after 18 h, which was accompanied by robust 3-HP production. This result indicated higher activity of the ECFHPG strain. Indeed, while the total biomass decreased, the abundance of the ECFHPG strain increased by 2.2-fold (40.4%) compared to that in the absence of ampicillin at 48 h (Fig. 4h). It was also observed that the C-mole yield of 3-HP increased by 4.38-fold compared to the case where ampicillin was not added (Supplementary Table 2), showing that the population control strategy facilitated the successful acclimation of the population for improvement of 3-HP production.

We further applied a higher level of the selection pressure by increasing the concentration of ampicillin to 20 µg/mL to determine whether 3-HP production could be further increased. In response to this alteration, the growth of the consortium was inhibited significantly; only 2.53 g/L of alginate was consumed, which resulted in low biomass formation (final OD_600_ value: 5.30) (Fig. 4d). Probably due to the very small of alginate consumption and biomass formation, the acetate levels also remained low (below 0.23 g/L) throughout the fermentation period. Nevertheless, even at such high selection pressure, 199.40 mg/L of 3-HP was produced with the highest yield (Supplementary Table 2). However, 20 µg/mL of ampicillin appeared to be excessively high, given that 3-HP was produced only during the initial 12 h at a lower titer than that obtained with 10 µg/mL of ampicillin (293.55 mg/L). The 3-HP production was stalled probably due to unviable VDHG cells. The population analysis further confirmed the hypothesis that the production of 3-HP during the initial phase of culturing resulted from the increased population ratio of ECFHPG.

### Comparison with a 3-HP independent *bla* expression strategy

We compared this 3-HP-dependent *bla* expression strategy (i.e., conditional cooperation) with a static *bla* expression strategy (i.e., simple cooperation). For this comparison, we constructed the pC112-3HP, pC100-3HP, and pC119-3HP plasmids that express *bla* under one of four constitutive promoters with different strengths by more than 10^3^-fold (P_J23112_, P_J23100_, and P_J23119_, in the order of strengths)^46^. We co-cultured VDHG and ECFHPC1-3 (harboring each *bla* expression plasmid instead of the population guider, Supplementary Data 1) in the presence of 10 μg/mL of ampicillin (Supplementary Fig. 8). With the *bla* expression under P_J23119_, the highest amount of final biomass was achieved, indicating the greatest degradation of ampicillin. Notably, up to 168.00 mg/L of 3-HP was produced at 48 h, which was higher than that of the co-culture of the VDHG and ECFHPG strains without addition of ampicillin, indicating the simple cooperative interaction was also advantageous for 3-HP production. However, no significant correlation in 3-HP production and promoter strengths was observed. In addition, 1~2 g/L of acetate was consistently accumulated until 24 h, suggesting the suboptimal performance of the consortia compared with the results of the previous 3-HP-dependent *bla* expression strategy.

We utilized mathematical modeling (see Supplementary Note 2 and Supplementary Table 3) for better understanding and comparing the achieved results. Due to limitations to model a dynamically changing environment, we accounted alginate consumption and remaining acetate for estimating cell growth of both strains and 3-HP production under each condition. As observed, the construction of the synthetic cooperation was expected to yield higher 3-HP production over the simple co-cultivation (Supplementary Fig. 9). Moreover, a superior production with the 3-HP dependent *bla* expression to that with static *bla* expression was expected. The strong expression of *bla* led to the outgrowth of VDHG, resulting in a similar profile from the simple co-culture; 3-HP production was expected to be low even with the low expression, likely owing to insufficient growth of both microorganisms. Collectively, we concluded that the 3-HP dependent *bla* expression strategy can efficiently improve the 3-HP production by the co-culture of *Vibrio* sp. dhg and 3-HP producing *E. coli* strains.

## Discussion

In this study, we demonstrated the efficient conversion of alginate to 3-HP by constructing a microbial consortium of alginate-metabolizing *Vibrio* sp. dhg and 3-HP-producing *E. coli* strains and introducing a population-guiding genetic circuit to acclimatize their populations for improving 3-HP production. Although simple co-cultivation of the strains also led to the production of 3-HP from alginate, the dominance of *Vibrio* sp. dhg in the culture resulted in the high accumulation of acetate, which is a pathway intermediate, and the levels of 3-HP production were also low. From the observation that the population ratio significantly affected the biochemical production efficiencies, we introduced the population-guiding circuit and selection pressure to establish cooperative interaction between the strains. Consequently, acclimated consortium population led to a significant improvement in 3-HP production from alginate.

The results clearly indicate that synthetic microbial consortia can serve as a powerful platform for biochemical production from non-conventional substrates. Although several successful attempts have been made, the engineering of microorganisms that can utilize non-conventional substrates is mostly difficult owing to insufficient genetic information and molecular tools. However, co-culturing with microorganisms with known genetic information can help overcome the intrinsic limitations of the single-cell engineering approach. As shown in this study, a synthetic consortium can leverage the useful properties of each microorganism to develop a novel pathway or function that does not exist in a single microorganism, and resultantly, synthesize value-added biochemical products successfully.

To leverage the huge potential of the co-culture approach, efficient population control strategy needs to be devised. However, despite its potential, only limited strategies (e.g., programmed cell lysis depending on the level of a quorum sensing molecule) for optimizing the composition of consortia have been proposed and applied for biochemical production. Although previous approaches successfully demonstrated that a microbial population is controllable to some extent, since we still do not know what composition is optimal for biochemical production during dynamically changing fermentation environment, the application of these strategies in bioproduction can be challenging. On the contrary, a “population guider” was designed to directly associate the “survival” of cells with biochemical production and to find an optimal population by autonomous acclimation of the population toward improved production of a target.

Despite the success, there are several issues for broad applications of our approach. (i) This strategy was applied to a two-party bacterial consortium where only one strain can utilize the substrate and the other strain relies on the byproduct formation. However, co-culturing two different strains are very relevant for bioprocesses, especially for the conversion of non-conventional feedstocks. In addition, acclimation of a consortium with more than two members can be also attempted if one finds a suitable pair of a growth inhibitor and its degradation gene to construct a “conditional cooperation” based on target chemical production. (ii) The requirement of antibiotics itself or potential difference in antibiotic susceptibility of each party can act as a significant constraint^47,48^. We expect that ampicillin can be replaced by any growth inhibitors which can commonly inhibit consortium participants. The use of antibiotics can be avoided by directly engineering cells to produce growth-inhibiting compounds (e.g., bacteriocins)^49,50^. For example, microcin S (encoded by *mcsS*) from probiotic *E. coli* G3/10 is known to inhibit the growth of neighboring microorganisms^50^. The population would be similarly acclimated for higher production if a gene (e.g., *mcsI*) for degrading microcin S is expressed or the expression of *mcsS* is reduced when a target chemical is produced. Furthermore, in the case that consortium participants show different extent of susceptibility, multiple growth inhibitors specifically effecting a single strain can be also utilized. (iii) To build a “population guider” for the production of other chemicals, a molecular part that can detect a target chemical must be available. However, with growing interest in developing synthetic circuits which can process diverse inputs, the capability of excavating or developing protein- and RNA-biosensors has been extensively improved. For example, parts were newly found or designed for small metabolites^51,52^ and natural products^53^. Thus, we foresee that the “molecular catalog” of biosensors is more expanded and widely available. (iv) The choice of the level of the selection pressure also plays a critical role and thus it needs an optimization. There was room for further optimization of the ampicillin condition between 10 and 20 μg/mL (Fig. 4). And during a separate repeated culture, it seemed that the level also affects the degradation of 3-HP after the depletion of available carbon source (Supplementary Fig. 10). Therefore, to achieve even higher production, a detailed follow-up study about an optimal strategy to apply the selection pressure needs to be carried out.

One still may question “Is a consortium approach still needed even if genetic engineering of a non-model host becomes readily available?”. Indeed, genetic engineering of *Vibrio* species has been recently studied^35,54,55^ to leverage their rapid growth and high metabolic capacities. Therefore, it is expected that direct conversion of 3-HP from alginate using a single strain will be available soon. Although there is no clear answer, as mentioned in the introduction section, the consortium approach can be more efficient since it allows division of labor, simplification of a complex metabolic pathway^4^. However, since a population control is the key to develop a successful multi-strain bioprocess, diverse strategies should be devised and tested. In this regard, we believe that a production-dependent synthetic cooperation can be an effective strategy as shown with the current model consortium of *Vibrio* sp. dhg and *E. coli*.

Collectively, this study shows the circuit-based population-guiding strategy is a promising tool for the development of efficient consortium-based bioprocesses. The high flexibility in the design of a microbial consortium can facilitates the development of novel processes to produce diverse biochemicals from nonconventional feedstocks by employing multiple modularized strains.

## Methods

### Bacterial strains, plasmids, and reagents

The bacterial strains and plasmids used are listed in Supplementary Data 1. Methods for plasmid construction are elaborated in Supplementary Note 3. The primers were synthesized by Cosmogenetech (Seoul, Korea) and are listed in Supplementary Data 2. Plasmid DNA was isolated using an Exprep^TM^ Plasmid SV kit from GeneAll (Seoul, Korea). DNA fragments amplified by PCR were purified using an Expin^TM^ Gel SV kit (GeneAll). Q5 polymerase and restriction enzymes were purchased from New England Biolabs (Ipswich, MA, USA). The reagents for cell cultures were purchased from BD Bioscience (Sparks, MD, USA). All other chemicals were obtained from Sigma (St. Louis, MO, USA) as listed in Supplementary Data 2, unless otherwise indicated.

### Culture conditions for *Vibrio* sp. dhg and *E. coli* strains

All flask-scale cell cultures were performed using 300-mL flasks containing 50 mL of a modified minimal medium^34^. The medium consisted of 15 g/L NaCl, 5 g/L (NH_4_)_2_SO_4_, 0.5 g/L MgSO_4_·7 H_2_O, 100 mM potassium phosphate buffer (pH 7), and 2 mL/L trace metal solution (ATCC MD-TMS, pH 7.0). The trace metal solution contained 0.5 g/L ethylenediaminetetraacetic acid (EDTA), 3.0 g/L MgSO_4_·H_2_O, 0.5 g/L MnSO_4_·H_2_O, 1.0 g/L NaCl, 0.1 g/L FeSO_4_·7H_2_O, 0.1 g/L Co(NO_3_)_2_·6 H_2_O, 0.1 g/L CaCl_2_, 0.1 g/L ZnSO_4_·7H_2_O, 0.01 g/L CuSO_4_·5H_2_O, 0.01 g/L AlK(SO_4_)_2_, 0.01 g/L H_3_BO_3_, 0.01 g/L Na_2_MoO_4_·2 H_2_O, 0.001 g/L Na_2_SeO_3_, 0.01 g/L Na_2_WO_4_·2 H_2_O, and 0.02 g/L NiCl_2_·6 H_2_O. The concentration of NaCl in the medium was adjusted to 15 g/L for the co-culture of *Vibrio* sp. dhg and *E. coli* strains. Alginate or acetate (neutralized to pH 7 by the addition of NaOH) was provided as a carbon source depending on the experimental conditions. All cell culture experiments were performed at 30 °C with continuous shaking at 200 rpm.

Seed cultures of *Vibrio* sp. dhg were prepared by inoculating a colony into minimal medium supplemented with 10 g/L alginate. In case of *E. coli* strains, minimal medium supplemented with 1 g/L yeast extract and 4 g/L acetate was used. After overnight culture, the cells were refreshed by inoculating in fresh media without yeast extract at an OD_600_ of 0.05. After the refreshed cells were cultured till an OD_600_ value of 1.0 was achieved, the microorganisms were co-cultured at OD_600_ 0.5 and 2 for *Vibrio* sp. dhg and *E. coli*, respectively. For 3-HP production, isopropyl β-D-1-thiogalactopyranoside was added to a final concentration of 0.1 mM in both *E. coli* refreshing and co-cultures. Depending on the experimental conditions, various quantities of 3-HP (0, 0.5, and 2 g/L) or ampicillin (0, 1, 2, 5, 10, and 20 μg/mL) were added. For the stable maintenance of plasmids, streptomycin (50 µg/mL) or chloramphenicol (10 µg/mL) was added to the media.

### Construction of the population guider and its testing

To construct the population guider, we generated two 5’-UTR variants (Supplementary Table 1) with different predicted expression levels of 22,884 and 115,863 using the 5’-UTR Library Designer^56^. The two plasmids with each 5’-UTR of *bla* (pPopG1 and pPopG2 plasmids) were introduced into the *E. coli* W strain, which led to the formation of the ECPG1 and ECPG2 strains. For testing the population guider, the cultures were performed on a small scale (180 μL as culture volume) using a BioscreenC MBR (Oy Growth Curves Ab, Helsinki, Finland). Seed cultures were prepared using the method used for flask-scale cultures. Refreshed seed cultures were diluted in fresh media at an OD_600_ of 0.05 and supplemented with ampicillin (50 μg/mL) and 3-HP (0, 0.5, and 2 g/L). The growth of the cultures (in terms of OD_600_ value) was monitored for 24 h at 30 °C under normal shaking with high amplitude. Three biological replicates of the small-scale cell cultures were used.

### Metabolite quantification

Alginate was quantified using a previously reported protocol^34,57^. Briefly, 200 μL of each sample was mixed with 1 mL of 0.025 M Na_2_B_4_O_7_·10H_2_O in H_2_SO_4_ (95–98% v/v). After cooling the mixture to 0 °C, 40 μL of 0.125% (w/v) carbazole dissolved in absolute ethanol was added, and the mixture was shaken gently. The absorbance was measured at 530 nm using a Hidex Sense microplate reader (Hidex, Turku, Finland). A standard curve was generated using alginate samples with concentrations in the range of 0-2 g/L. For calculation of fermentation parameters shown in Supplementary Table 2, alginate concentrations at 48 h were utilized.

Acetate and 3-HP were quantified using an UltiMate 3000 analytical HPLC system (Dionex, Sunnyvale, CA, USA) equipped with an Aminex HPX-87H column (Bio-Rad Laboratories, Richmond, CA, USA). For the mobile phase, 5 mM sulfuric acid was used at a flow rate of 0.6 mL/min. The temperature of the column compartment was maintained at 14 °C. Acetate and 3-HP signals were monitored using a Shodex RI-101 refractive index detector (Shodex, Klokkerfaldet, Denmark).

### Quantification of the population ratio of *Vibrio* sp. dhg and *E. coli*

The population ratios of *Vibrio* sp. dhg (expressing *mcherry*) and *E. coli* (expressing *sgfp*) were determined by counting the number of cells of each strain emitting fluorescence using a Beckman Coulter flow cytometer (Brea, CA, United States). The cultured samples were washed using fresh minimal medium and injected into the flow cytometer after their OD_600_ value was adjusted to 1 (corresponding to 10^6^ cells/μL). mCherry fluorescence was detected using a 488-nm excitation laser and 610/20-nm emission channel with an energy-coupled dye bandpass filter. sGFP fluorescence was detected using a 488-nm excitation laser and a 525/40-nm emission channel with a fluorescein isothiocyanate (FITC) bandpass filter. For each sample, the fluorescence signals of at least 100,000 cells were monitored. The ratio of *Vibrio* sp. dhg and *E. coli* cells was calculated by dividing each fluorescent cell number value by the total cell number value. A standard curve was generated using known ratios of *Vibrio* sp. dhg and *E. coli* (Supplementary Fig. 2).

### Statistical analysis

Statistical tests were performed using the two-tailed Student’s t-test by SigmaPlot (SigmaStat).

### Reporting summary

Further information on research design is available in the Nature Research Reporting Summary linked to this article.

## Supplementary information

Supplementary Information

Description of Additional Supplementary Files

Reporting Summary

Supplementary Data 1

Supplementary Data 2

## Data Availability

A reporting summary for this article is available as a Supplementary Information file. The strains and plasmids used in this study are provided as a Supplementary Data 1 file. The primers and reagents information used in this study are provided as a Supplementary Data 2 file. Synthetic promoter sequences were obtained from Registry of Standard Biological Parts (http://parts.igem.org/Main_Page). The raw data for modeling growth of *Vibrio* sp. dhg and *E. coli* strains are accessible at 10.5281/zenodo.7187480. Source data are provided with this paper.

## Source data

Source Data

## Code availability

Computational code is accessible at 10.5281/zenodo.7187480.
