# Supplementary material for: Circuit-guided population acclimation of a synthetic microbial consortium for improved biochemical production
Source: Nat Commun. 2022 Nov 7;13:6506. doi: 10.1038/s41467-022-34190-z (PMC9640620; doi:10.1038/s41467-022-34190-z)
Supplement: Supplementary file 1 — Supplementary Information [file 41467_2022_34190_MOESM1_ESM.docx]

Supplementary Information for
Circuit-guided acclimation of a synthetic bacterial consortium for improved conversion of alginate to a product

Chae Won Kang^1,a^, Hyun Gyu Lim^1,a^, Jaehyuk Won^2,3,a^, Sanghak Cha^1^, Giyoung Shin^4^, Jae-Seong Yang^5^, Jaeyoung Sung^2,3^, Gyoo Yeol Jung^1,4,*^

^1^Department of Chemical Engineering, Pohang University of Science and Technology, 77 Cheongam-Ro, Nam-Gu, Pohang, Gyeongbuk 37673, Korea

^2^Creative Research Initiative Center for Chemical Dynamics in Living Cells, Chung-Ang University, 84 Heukseok-Ro, Dongjak-gu, Seoul, 06974, Republic of Korea

^3^Department of Chemistry, Chung-Ang University, 84 Heukseok-Ro, Dongjak-gu, Seoul, 06974, Republic of Korea

^4^School of Interdisciplinary Bioscience and Bioengineering, Pohang University of Science and Technology, 77 Cheongam-Ro, Nam-Gu, Pohang, Gyeongbuk 37673, Korea.

^5^Centre for Research in Agricultural Genomics (CRAG), CSIC-IRTA-UAB-UB, Campus UAB, Bellaterra, Barcelona, 08193, Spain

^a^These authors contributed equally.

*Corresponding author:

Gyoo Yeol Jung

Mailing address: Department of Chemical Engineering, Pohang University of Science and Technology, 77 Cheongam-Ro, Nam-Gu, Pohang, Gyeongbuk 37673, Korea

Tel.: +82-54-279-2391, Fax: +82-54-279-5528, E-mail: gyjung@postech.ac.kr

# Supplementary Note

## Supplementary Note 1. Genetic engineering of *E. coli* W for 3-hydroxypropionic acid (3-HP) production from acetate

3-HP can be produced from malonyl-CoA using malonyl-CoA reductase (Mcr) from *Chloroflexus aurantiacus* with malonate semialdehyde as an intermediate product (Supplementary Fig. 1a)[^1^](https://sciwheel.com/work/citation?ids=3263058&pre=&suf=&sa=0&dbf=0). Mcr is a fusion protein of a reductase (the N-terminal part of Mcr, Mcr-N) and dehydrogenase (the C-terminal part of Mcr, Mcr-C). In a previous study[^2^](https://sciwheel.com/work/citation?ids=5053098&pre=&suf=&sa=0&dbf=0), it was observed that 3-HP production improved in response to the introduction of three base alterations (N940V, K1106W, and S1114R) in Mcr, along with the control of its expression level after division of the gene into two separate sections. Furthermore, in another study, the blockade of the metabolic flux to fatty acid production increased the levels of malonyl-CoA available for 3-HP production, thereby increasing the 3-HP titer[^3^](https://sciwheel.com/work/citation?ids=6018283&pre=&suf=&sa=0&dbf=0).

Based on findings from previous studies, we engineered the malonyl-CoA-dependent 3-HP producing pathway in *E. coli* to facilitate its production (at high levels) from acetate (Supplementary Fig. 1b). The mutant *mcr* gene was divided into the *mcr-n* and *mcr-c* genes, and each gene was overexpressed using a strong inducible promoter (P_tac_) and a synthetic 5’-UTR (Supplementary Table 1) to increase the carbon flux toward 3-HP biosynthesis. In addition, the malonyl-CoA node between fatty acid production and 3-HP production was optimized by altering the expression of the *fabHDG* operon. To achieve this optimization, we replaced the promoter of the *fabHDG* operon with a promoter library of degenerated -35 and -10 boxes of the P_J23100_ promoter (5’-YTKAYRGCTAGCTCAGTCCTAGGKAYWRTGCTAGC-3’). This introduction led to the formation of colonies of different sizes owing to the reduced metabolic flux to fatty acid synthesis, which is an essential component of cell growth. After obtaining six colonies of different sizes, we transformed the cells with pPopG1-3HP (plasmid harboring the *mcr-c* and *mcr-n* fragments for production of 3-HP and 3-HP-responsive genetic circuits) and compared the 3-HP titers produced. We observed that the 3-HP titer produced by strain 6 was the highest (272.82 mg/L from 10 g/L acetate), and showed a 5.74-fold increase compared to the titer produced by the wild-type strain during a 48-h fermentation period (Supplementary Fig. 1c). The promoter sequence of the *fabHDG* operon in strain 6 was 5’-TTGATGGCTAGCTCAGTCCTAGGGATAGTGCTAGC-3’; we named this strain ECF (Supplementary Data 1).

## Supplementary Note 2. Modeling of the synthetic microbial consortium system

Mathematical model framework

To explain the population dynamics of the consortium, we developed a theoretical model describing the interaction between *Vibrio* sp. dhg and *E. coli*.

Consider a simple model where the population grows via nutrient consumption:

$Y_{A/N}\Delta S_{N}=\Delta X_{A}$

where $X_{A}$ is the cell density of strain A, $S_{N}$ is the concentration of nutrient $N$, and $Y_{A/N}$ is the biomass yield. By differentiating Eq. S1, the uptake rate $r_{N/A}(t)$ of nutrient $N$ by strain A can be obtained as

$r_{N/A}\left( t \right)=-\frac{dS_{N}(t)}{dt}=\frac{1}{Y_{A/N}}\frac{dX_{A}(t)}{dt}$

Cell lysis and death can be approximated to the first order kinetics using the basal death rate $k_{A}^{d}$ of strain $A$. The model for the single bacterial strain $A$ can be written as

$\frac{dX_{A}(t)}{dt}=Y_{A/N}r_{N/A}(t)-k_{A}^{d}X_{A}(t)$

During the co-culture, growth limiting reagents are alginate and acetate, for *Vibrio* sp. dhg and *E. coli*, respectively. *Vibrio* sp. dhg assimilates acetate when alginate is depleted (t_d_). Additionally, the inhibitory effect of ampicillin to *Vibrio* sp. dhg was considered by using a Hill function[^4^](https://sciwheel.com/work/citation?ids=171836&pre=&suf=&sa=0&dbf=0). Therefore, the growth model for *Vibrio* sp. dhg and *E. coli* can be written as

$\frac{dX_{Vib}\left( t \right)}{dt}=Y_{Vib/Alg}r_{Alg/Vib}\left( t \right)+Y_{Vib/Ace}r_{Ace/Vib}\left( t \right)\theta\left( t-t_{d} \right)-(k_{Vib}^{d}+\mu_{Vib/Amp}^{d}\frac{S_{Amp}(t)}{K_{Vib/Amp}^{d}+S_{Amp}(t)})X_{Vib}(t)$

$\frac{dX_{Ecoli}(t)}{dt}=Y_{Ecoli/Ace}r_{Ace/Ecoli}(t)-k_{Ecoli}^{d}(t)X_{Ecoli}(t)$

Furthermore, since only *Vibrio* sp. dhg metabolizes alginate and produces acetate as a metabolite while *E. coli* subsequently produces 3-HP by consuming acetate, production of acetate and 3-HP can be modeled using the uptake rate and the yield of nutrients.

$\frac{dS_{Ace}(t)}{dt}=Y_{Ace/Alg}r_{Alg/Vib}\left( t \right)-r_{Ace/Ecoli}\left( t \right)-r_{Ace/Vib}(t)\theta\left( t-t_{d} \right)$

$\frac{dS_{3-HP}(t)}{dt}=Y_{3-HP/Ace}r_{Ace/Ecoli}(t)$

where $Y_{Ace/Alg}$, $Y_{3-HP/Ace}$ denotes the yield parameters of acetate from alginate and 3-HP from acetate.

To consider the 3-HP dependent *bla* expression and subsequent degradation of ampicillin, we also utilized a Hill function.

$\frac{d}{dt}S_{Amp}(t)=-\mu_{Amp}^{d}\frac{S_{Amp}(t)}{K_{Amp}^{d}+S_{Amp}(t)}S_{3-HP}(t)$

where $K_{Amp}^{d}$ is the degradation rate of ampicillin.

For consortia with the constitutive expression of *bla*, Eq. S8 can be modified to

$\frac{d}{dt}S_{Amp}(t)=-{\mu'}_{Amp}^{d}\frac{S_{Amp}(t)}{K_{Amp}^{d}+S_{Amp}(t)}X_{Ecoli}(t)$

Parameter Fitting

Model parameters were obtained by using Mathematica. Differential equations were solved by using Mathematica’s ParametricNDsolve routine and Findfit routine was used for assisting experimental data fitting. To find the best-fitted parameters for the data, we used single-strain culture data. We obtained two basal parameters $Y,k^{d}$of each strain from the culture at ampicillin 0 µg/mL. Basal death rates were determined from the nutrient depletion condition (36 - 48 h). Yield parameters of acetate and 3-HP from alginate and acetate were also extracted from the culture data. Alginate uptake rates were obtained by differentiating the consumed alginate profile. The alginate uptake rate was estimated by using $\sum_{i} a_{i}t^{i-1}/\sum_{j} b_{j}t^{j-1}$. We defined a depletion time of alginate ($t_{d})$ as the time where the alginate concentration becomes less than 2 g/L after 6 h in each experiment.

To quantitatively analyze the efficiency of *E. coli* consuming acetate, we used the Monod equation. The relation between nutrient and bacterial strain population growth can be modeled with the Monod equation[^5,6^](https://sciwheel.com/work/citation?ids=11339412,5499388&pre=&pre=&suf=&suf=&sa=0,0&dbf=0&dbf=0).

$r_{N}(t)=\frac{\mu_{A/N}}{Y_{A/N}}\frac{S_{N}(t)}{K_{A/N}+S_{N}(t)}X_{A}(t)$

The Monod parameters $\mu_{A/N}$ and $K_{A/N}$ denote the maximum growth rate and the half concentration respectively. By combining Eq. S6 and S10, Eq. S6 can be rewritten as the following equation:

$$\frac{dS_{Ace}(t)}{dt}=Y_{Ace/Alg}r_{Alg/Vib}(t)-\frac{\mu_{Ecoli/Ace}}{Y_{Ecoli/Ace}}\frac{S_{Ace}(t)}{K_{Ecoli/Ace}+S_{Ace}(t)}X_{Ecoli}(t)$$

$-\frac{\mu_{Vib/Ace}}{Y_{Vib/Ace}}\frac{S_{Ace}(t)}{K_{Vib/Ace}+S_{Ace}(t)}X_{Vib}(t)\theta(t-t_{d})$

The Monod parameters, $\mu_{Ecoli/Ace}$ , $K_{Ecoli/Ace}$, $\mu_{Vib/Ace}$ , and $K_{Vib/Ace}$were determined from the mono-culture profile of *E. coli* and *Vibrio* sp. dhg growing on acetate. The death rate of *Vibrio* sp. dhg by the effect of ampicillin was obtained from the co-culture profile with ampicillin 5 µg/mL. $\mu_{Ecoli/Ace}$ value was obtained from the culture without ampicillin addition. Other parameters for ampicillin degradation were obtained by fitting *Vibrio* sp. dhg profiles with three different concentrations of ampicillin (5, 10, 20 μg/mL). The obtained parameter values were provided in Supplementary Table 3.

## Supplementary Note 3. Plasmid construction

All plasmid manipulations were conducted using the Mach-T1^R^ strain (Supplementary Data 1) as a host. To construct the pCDF-sGFP plasmid, the sequence of the N-terminal-modified *sgfp* was amplified using the pCDF-106C4sgfp template[^7^](https://sciwheel.com/work/citation?ids=5200290&pre=&suf=&sa=0&dbf=0) with the O-sGFP-F1, O-sGFP-F2, O-sGFP-B1, and O-sGFP-B2 primers. The purified DNA fragments and pCDFDuet plasmid were digested using *Sac*I and ligated. Similarly, the pACYC-mCherry plasmid was constructed by insertion of DNA fragments amplified using the O-mCherry-F1, O-mCherry-F2, and O-mCherry-B primers with pZ-mCherry as a template into the *Sac*I site of pACYCDuet. To facilitate the high expression of the *sgfp* and *mcherry* reporter genes, we used a strong promoter (P_J23100_) and 5’-UTR sequences (Supplementary Table 1).

The pCDF-sGFP-MCR plasmid was constructed to express *mcr-c* and *mcr-n* independently at high levels. The *mcr-c* fragment was amplified using the O-MCRC-F1, O-MCRC-F2, and O-MCRC-B primers*,* and the *mcr-n* fragment was amplified using the O-MCRN-F1, O-MCRN-F2, and O-MCRN-G primers*.* The pET-*mcr** plasmid was used as a template for amplification. The genes were expressed under a strong inducible promoter (P_tac_) and synthetic 5’-UTRs for overexpression. The *mcr-c* and *mcr-n* fragments were inserted into pCDF-sGFP to produce the pCDF-sGFP-MCR plasmid using the *Sph*I, *Sac*I, and *Sal*I restriction sites.

In a previous study[^7^](https://sciwheel.com/work/citation?ids=5200290&pre=&suf=&sa=0&dbf=0), the pCDF-106C4sgfp plasmid was constructed to express *sgfp* depending on the titer of 3-HP using a 3-HP inducible transcription factor (C4-LysR) expressed under the P_J23106_ promoter and its cognate promoter from *Pseudomonas denitrificans.* For the 3-HP dependent expression of *bla*, the pPopG1 and pPopG2 plasmids were constructed by replacing *sgfp* from the pCDF-106C4sgfp plasmid. The plasmid fragment was amplified using the pCDF-106C4sgfp plasmid and the O-LysRP_C4M_-F and O-LysRP_C4M_-B primers. The *bla* gene was amplified using the pETDuet plasmid as a template and the O-bla-Fv1, O-bla-Fv2, and O-bla-B primers. The purified DNA fragments were digested using *Mfe*I and *Hind*III and ligated to construct the pPopG1 and pPopG2 plasmids. Finally, pPopG was constructed by inserting the *sgfp* cassette into the *Sac*I cloning site of the pPopG1 plasmid. Next, the pPopG-3HP plasmid was constructed by assembling the amplified *mcr-c* and *mcr-n* constructs using the O-MCRC-F2 and O-MCRN-B primers with the pCDF-sGFP-MCR plasmid into the *Sph*I and *Sal*I sites of the pPopG plasmid.

The pC112-3HP, pC100-3HP, and pC119-3HP plasmids were constructed to constitutively express *bla* gene independent of 3-HP. The *bla* gene was amplified using the pETDuet plasmid as a template and the O-Cbla-Fv1, O-Cbla-Fv2, O-Cbla-Fv3, and O-Cbla-B primers. The genes were expressed under three synthetic promoters (P_J23112_, P_J23100_, and P_J23119_) and synthetic 5’-UTR for constitutive expression at different expression levels. The amplified gene fragments were inserted into the *Hind*III cloning site of the pCDF-sGFP-MCR plasmid to produce pC112-3HP, pC100-3HP, and pC119-3HP plasmids.

Of note, the synthetic constitutive promoter and terminator (BBa_B1002) sequence was obtained from the Registry of Standard Biological Parts (<http://parts.igem.org/>). In addition, the 5’-UTR sequences were designed using UTR Designer (<http://sbi.postech.ac.kr/rbs>)[^8^](https://sciwheel.com/work/citation?ids=1239735&pre=&suf=&sa=0&dbf=0).

# Supplementary Tables

## Supplementary Table 1. 5’-UTR sequences used in this study

| **Plasmid name** | **Gene symbol** | **Predicted expression level** | **5’-UTR sequence (5’-3’)^*^** |
| --- | --- | --- | --- |
| pPopG1 | *bla* | 22,884  (weak) | attgacgccgaaagtcggatcagag |
| pPopG2 | *bla* | 115,863  (strong) | attgacgccgaaaggagcatcagag |
| pPopG-3HP | *mcr-c* | 1,105,318  (strong) | aacaattactagtaaggaggaaaga |
|  | *mcr-n* | 396,578  (strong) | ggataacaattaaggagcactgtac |

^*^The 5’-UTR sequences were designed using UTR Designer[^8^](https://sciwheel.com/work/citation?ids=1239735&pre=&suf=&sa=0&dbf=0).

## Supplementary Table 2. Comparison of microbial consortia developed in this study^a^

| **Ampicillin (µg/mL)** | **Consumed**  **alginate (g/L)** | **Final Biomass** | **Final population ratio (%)^b^** | | **3-HP (mg/L)** | **Maximum accumulated acetate (g/L)** | **3-HP productivity**  **(mg/L/h)** | **3-HP yield**  **(mg/g)** | **Overall**  **C-mole yield (%)** |
| --- | --- | --- | --- | --- | --- | --- | --- | --- | --- |
|  |  |  | **VDHG** | **ECFHPG** |  |  |  |  |  |
| 0 | 19.58  ± 0.25  (100%) | 18.70  ± 1.57  (29.73%) | 81.50  ± 1.38  (26.75%) | 18.50  ± 1.38  (2.98%) | 68.35 ± 32.75  (0.37%) | 2.21  ± 0.20 | 1.42  ± 0.68 | 3.47  ± 1.63 | 30.09  ± 3.12 |
| 5 | 19.19  ± 0.07  (100%) | 16.29  ± 0.40  (26.12%) | 70.85  ± 0.10  (20.24%) | 29.15  ± 0.10  (5.88%) | 125.40  ± 39.50  (0.70%) | 1.44  ± 0.04 | 2.61  ± 0.82 | 6.53  ± 2.03 | 26.81  ± 0.66 |
| 10 | 19.37  ± 0.11  (100%) | 13.99  ± 0.75  (22.31%) | 59.57  ± 1.64  (14.16%) | 40.43  ± 1.64  (8.15%) | 293.55  ± 11.15  (1.62%) | 1.05  ± 0.03 | 6.12  ± 0.23 | 15.15  ± 0.49 | 23.93  ± 1.62 |
| 20 | 2.53  ± 0.29  (100%) | 5.30  ± 0.29  (40.42%) | 45.61  ± 4.83  (24.16%) | 54.39  ± 4.83  (16.26%) | 199.40  ± 2.60  (8.55%) | 0.23  ± 0.00 | 4.15  ± 0.05 | 79.74  ± 2.20 | 48.96  ± 0.49 |

^a^Values in parentheses show the C-mole percentage produced from consumed alginate.

^b^Population ratio is the value measured at 48 h. OD_600_ of 1 was assumed to be equivalent to 0.27 g and 0.31 g dry cell weight (DCW) per liter for *Vibrio* sp. dhg and *E. coli*, respectively[^9^](https://sciwheel.com/work/citation?ids=13419155&pre=&suf=&sa=0&dbf=0).

## Supplementary Table 3. Parameters and fitted values for modeling

| Parameter | Definition | Fitted value |
| --- | --- | --- |
| $Y_{Vib/Alg}$ | Biomass yield of *Vibrio* sp. dhg from alginate | 0.97 |
| $Y_{Vib/Ace}$ | Biomass yield of *Vibrio* sp. dhg from acetate | 0.90 |
| $Y_{Ecoli/Ace}$ | Biomass yield of *E. coli* from acetate | 1.24 |
| $k_{Vib}^{d}$ | Death rate of *Vibrio* sp. dhg | 0.0104 |
| $k_{Ecoli}^{d}$ | Death rate of *E. coli* | 0.013 |
| $Y_{Ace/Alg}$ | Acetate yield from alginate by *Vibrio* sp. dhg | 0.2 |
| $Y_{3-HP/Ace}$ | 3-HP yield from acetate by *E. coli* | 0.07 |
| $\mu_{Vib/Ace}$ | Maximum growth rate of *Vibrio* sp. dhg | 0.045 |
| $\mu_{Ecoli/Ace}$ | Maximum growth rate of *E. coli* | 0.0496 |
| $K_{Vib/Ace}$ | Half acetate concentration for *Vibrio* sp. dhg growth | 0.305 |
| $K_{Ecoli/Ace}$ | Half acetate concentration for *E. coli* growth | 0.305 |
| $\mu_{Vib/Amp}^{d}$ | Maximum death rate of *Vibrio* sp. dhg by ampicillin | 0.05 |
| $K_{Vib/Amp}^{d}$ | Half kill value of *Vibrio* sp. dhg by ampicillin | 5.0 |
| $\mu_{Amp}^{d}$ | Maximum degradation rate of ampicillin (3-HP dependent) | 4.0 |
| ${\mu^{'}}_{Amp}^{d}$ | Maximum degradation rate of ampicillin (P_J23119,_ P_J23112_) | 1.4, 0.52 |
| $K_{Amp}^{d}$ | Half degradation value of ampicillin | 5.0 |
| $X_{A}$ | Cell density of strain A |  |
| $S_{N}$ | Concentration of chemical, N |  |
| $r_{N}$ | Uptake rate of nutrient, N |  |

# Supplementary Figures


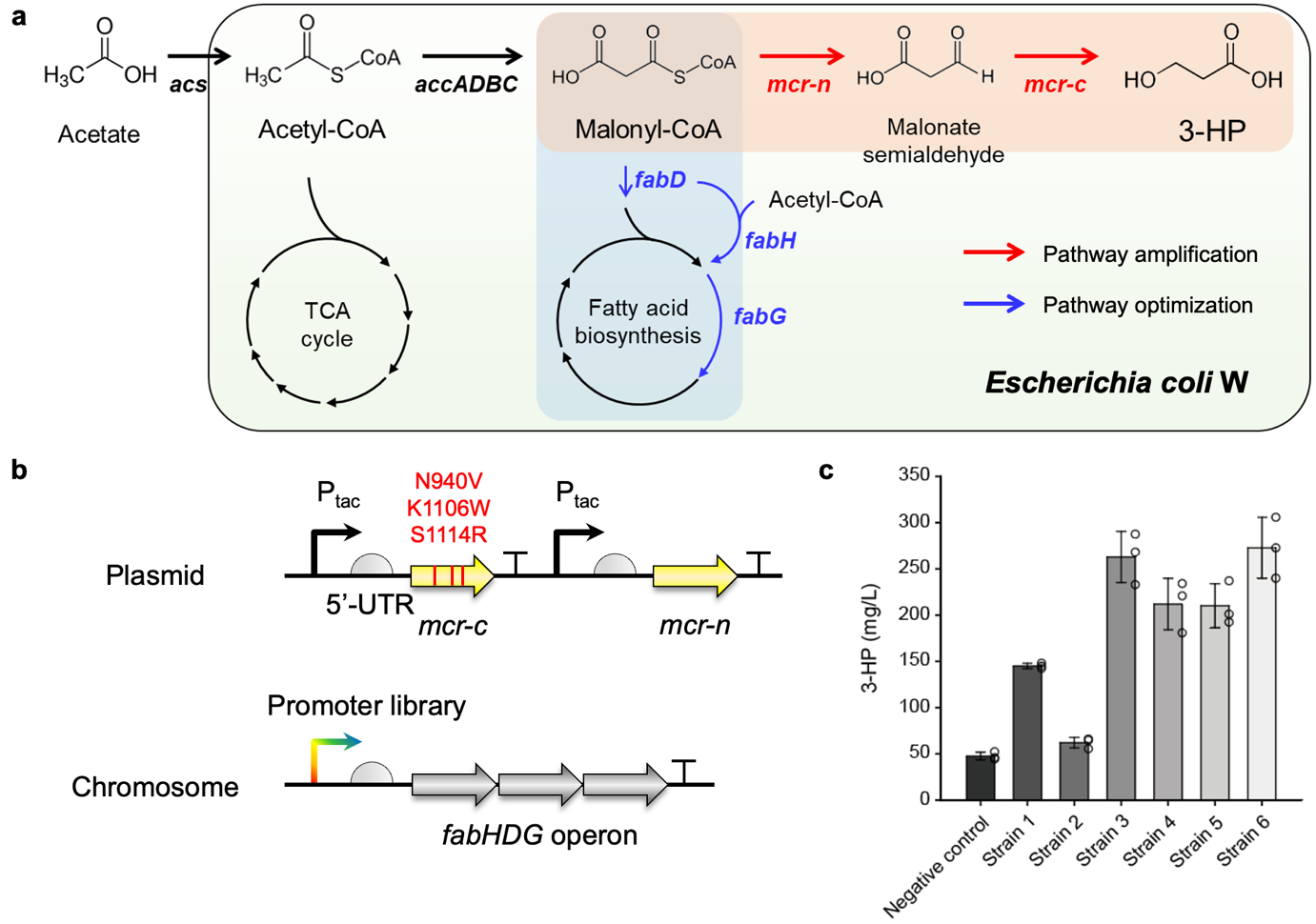


## **Supplementary Fig. 1 Engineered *E. coli* for efficient 3-HP production from acetate.**

**(a)** Malonyl-CoA-dependent pathway of 3-HP biosynthesis from acetate. *E. coli* converts acetate into acetyl-CoA using acetyl-CoA synthetase encoded by *acs*. Next, acetyl-CoA is converted to malonyl-CoA by acetyl-CoA carboxylase encoded by *accADBC*. Eventually, 3-HP is produced from malonyl-CoA by malonyl-CoA reductase encoded by *mcr-n* and *mcr-c* using malonate semialdehyde as an intermediate. The original *mcr* gene was dissected into two functional genes[^10^](https://sciwheel.com/work/citation?ids=5053101&pre=&suf=&sa=0&dbf=0). To achieve a high 3-HP titer, the *mcr-n* and *mcr-c* genes were overexpressed under P_tac_ and synthetic 5’-UTRs (Supplementary Table 1). *mcr-c* contains three mutations that leads to base alterations (N940V, K1106W, S1114R), which are known to improve the activity of the protein[^10^](https://sciwheel.com/work/citation?ids=5053101&pre=&suf=&sa=0&dbf=0). Furthermore, the metabolic flux toward fatty acid biosynthesis was controlled by diversifying the promoter sequences of the *fabHDG* operon. **(b)** Schematic diagram of gene expression cassette for *mcr-c*, *mcr-n,* and *fabHDG* genes. **(c)** Comparison of 3-HP titers produced by six different strains with mutant promoters of the *fabHDG* operon and the wild-type strain after 48-h fermentation. Strain 6 produced the highest 3-HP titer (272.82 mg/L from 10 g/L acetate). The *y*-axis represents 3-HP titer (mg/L), while the *x*-axis shows the variants with mutant promoters of *fabHDG*. Error bars represent standard deviations in triplicate biological experiments (*n* = 3) and their center indicates a mean value. Source data are provided as a Source Data file.


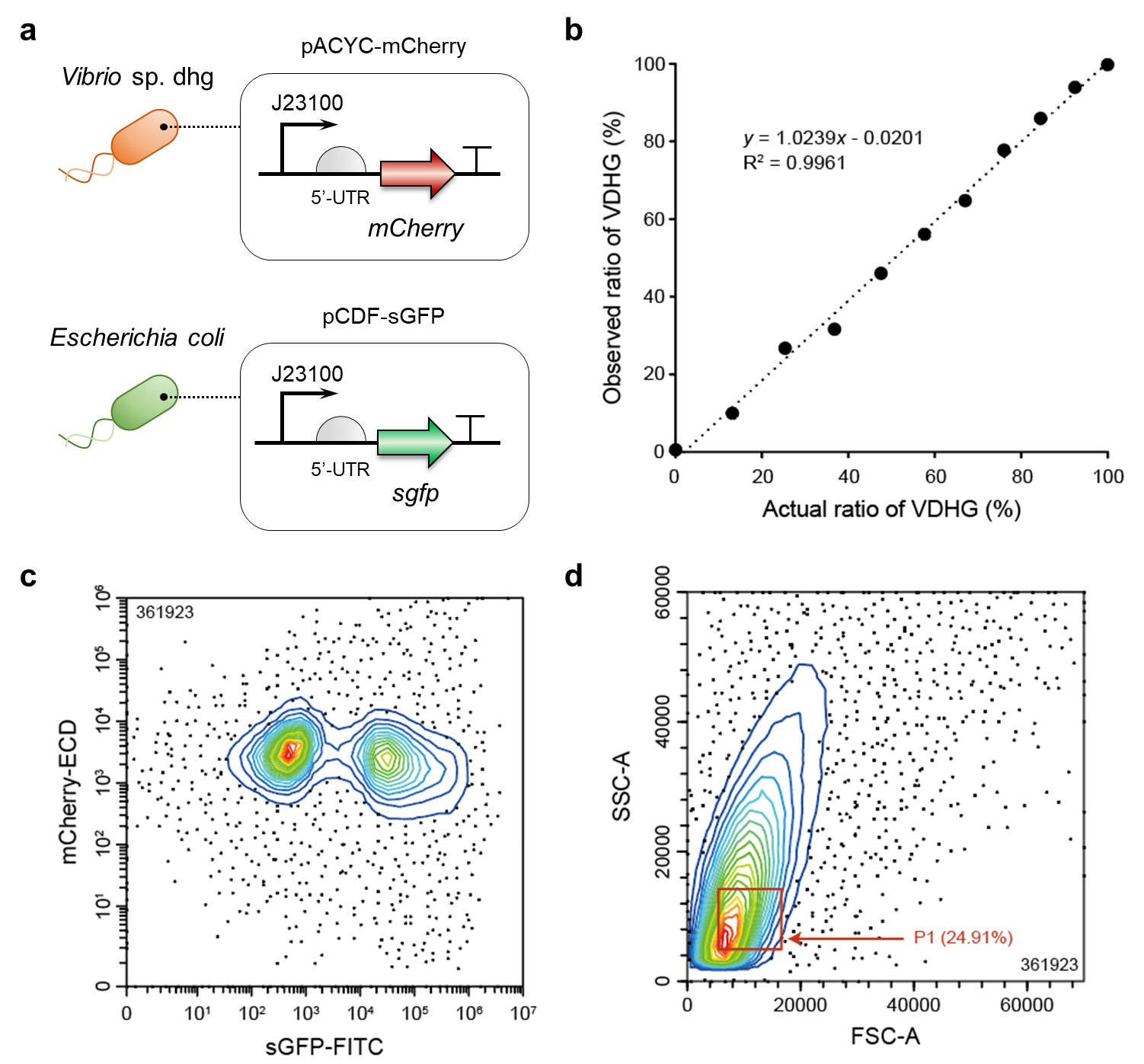


## Supplementary Fig. 2 Analysis of population ratios by fluorescent labeling of *Vibrio* sp. dhg and *E. coli* cells*.*

**(a)** The population ratio of *Vibrio* sp. dhg (VDHG) and *E. coli* (ECFHPG) strains measured by monitoring the fluorescence of each cell. *Vibrio* sp. dhg was fluorescently labeled by introducing the *mcherry* gene. *E. coli* was fluorescently labeled by introducing the *sgfp* gene. **(b)** Comparison of the actual and observed ratios of VDHG. After mixing VDHG and ECFHPG cells in known ratios, the population ratio was measured by flow cytometry. The *y*-axis and *x*-axis represent the actual and observed population ratios of VDHG, respectively. The correlation coefficient (R^2^) was 0.9961. **(c)** Contour plot of a sample in which VDHG and ECFHPG cells were mixed at a 1:1 ratio. The *y*-axis and *x*-axis represent the mCherry-ECD and sGFP-FITC, respectively. **(d)** Gating strategy of the flow cytometry data. For the analysis of flow cytometry data, the scatter plot for all events was gated (P1). The *y*-axis and *x*-axis represent the SSC-A and FSC-A, respectively. Source data are provided as a Source Data file.


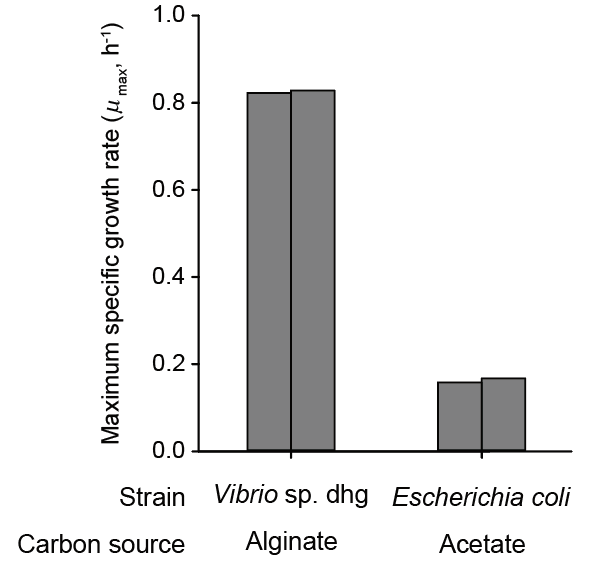


## Supplementary Fig. 3 Comparison of the maximum specific growth rates of *Vibrio* sp. dhg and *E. coli.*

Comparison of the maximum specific growth rates (*µ*_max_) of *Vibrio* sp. dhg (VDHG) in alginate and *E. coli* (ECPG1) in acetate. The *y*-axis represents the *µ*_max_ (h^-1^). Source data are provided as a Source Data file.


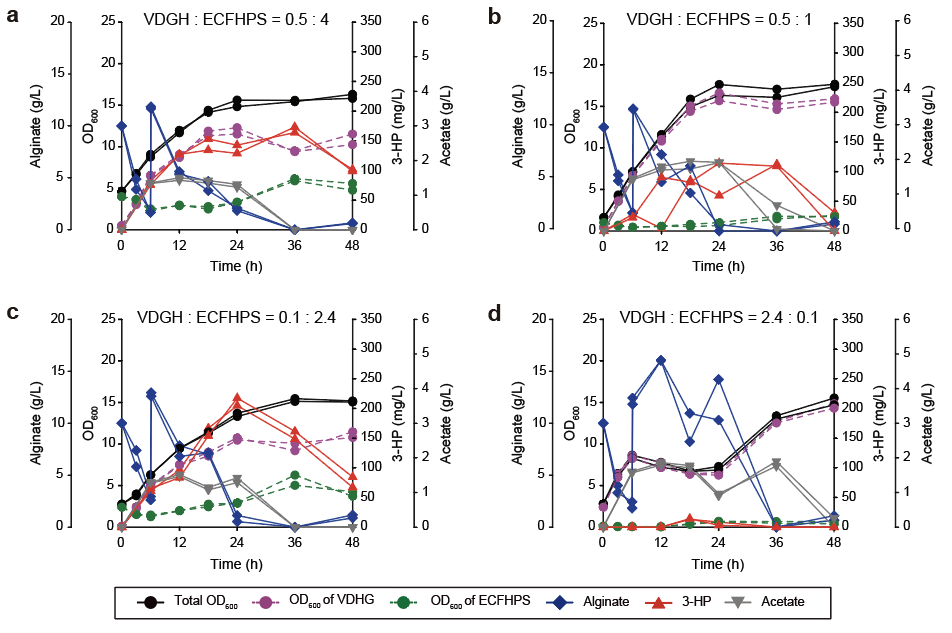


## Supplementary Fig. 4 Fermentation profile of the microbial consortium comprising VDHG and ECFHPS strains at various ratios.

**(a-d)** The fermentation profile of the co-culture of the VDHG and ECFHPS strains at the ratio of **(a)** 0.5 to 4, **(b)** 0.5 to 1, **(c)** 0.1 to 2.4 and **(d)** 2.4 to 0.1. The left *y*-axis represents OD_600_ of cultures (black circles). Estimated OD_600_ of the VDHG (purple circles) and ECFHPS (green circles) strains was also provided. The left *y*-offset, right *y*-axis, and right *y*-offset represent alginate (blue diamond, g/L), 3-HP (red triangles, mg/L) and acetate (grey inverted triangles, g/L) levels, respectively. Source data are provided as a Source Data file.


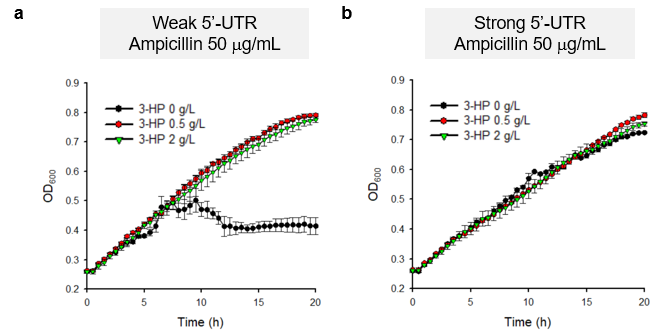


## Supplementary Fig. 5 Construction of a 3-HP-inducible genetic circuit.

Growth profiles of the **(a)** ECPG1 strain harboring the pPopG1 plasmid (weak 5’ UTR for *bla*, Supplementary Table 1), and the **(b)** ECPG2 strain harboring the pPopG2 plasmid (strong 5’ UTR for *bla*, Supplementary Table 1) in presence of 50 µg/mL of ampicillin. The genetic circuit was tested by adding various concentrations of 3-HP (0, 0.5, and 2 g/L). The *x*-axis and *y*-axis indicate the OD_600_ values and time (h), respectively. Error bars represent the standard deviations from triplicate biological experiments (*n* = 3) and their center indicates a mean value. Source data are provided as a Source Data file.


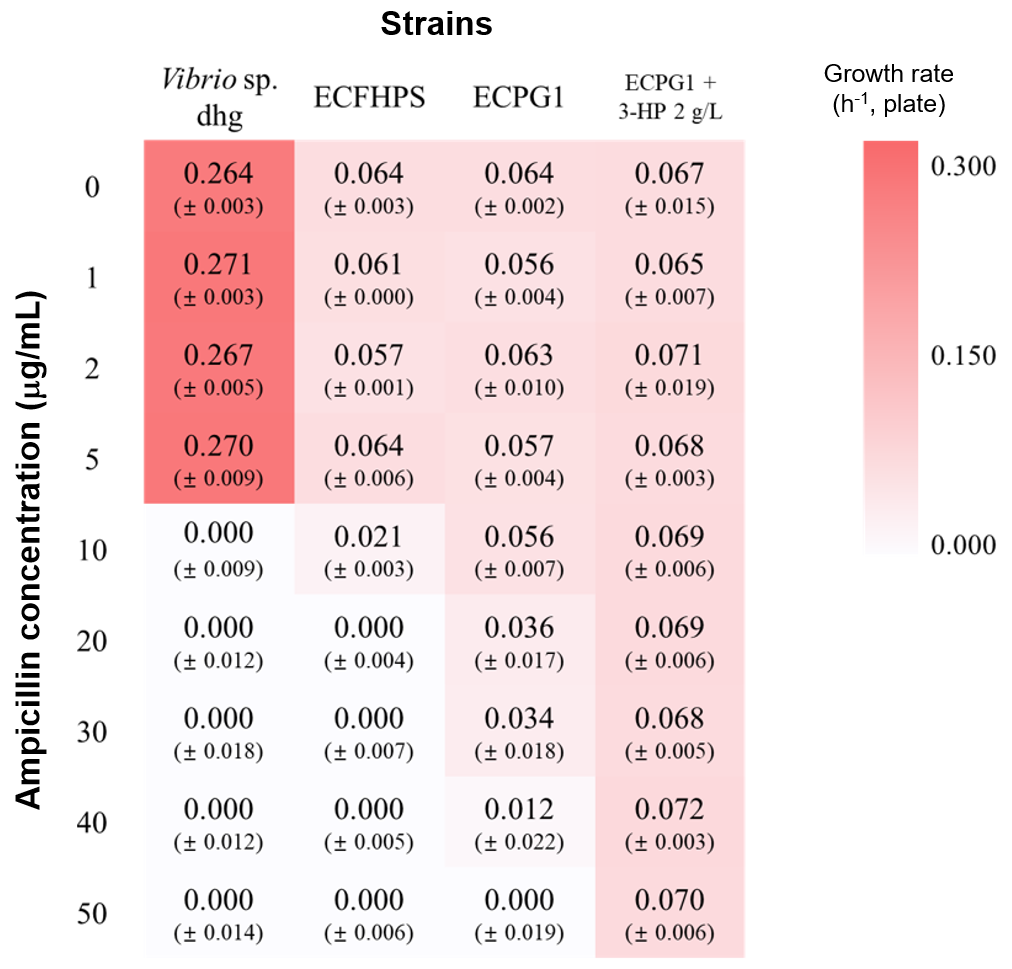


## Supplementary Fig. 6 Comparison of growth rates of *Vibrio* sp. dhg, ECFHPS, and ECPG1 strains at various ampicillin concentrations.

Growth rates (h^-1^) were measured in the presence of ampicillin at various concentrations from 0 to 50 μg/mL. Cells were growing in a 180 μL medium on a microtiter plate. Errors represent the standard deviations from triplicate biological experiments (*n* = 3) and the number above indicates a mean value. Source data are provided as a Source Data file.


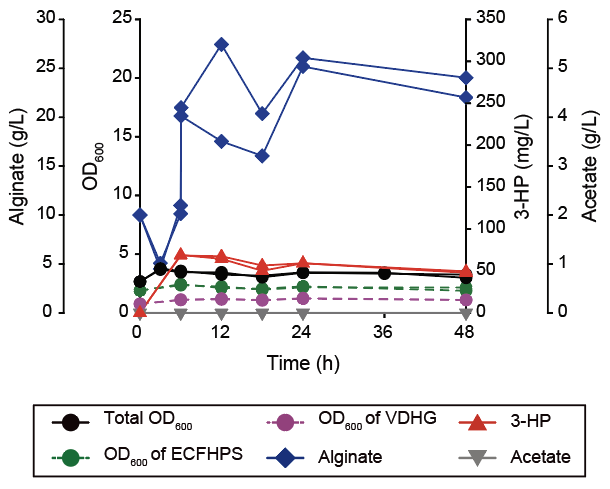


## Supplementary Fig. 7 Fermentation profile of the microbial consortium comprising VDHG and ECFHPS strains upon the addition of 10 µg/mL of ampicillin.

The fermentation profile of the co-culture of the VDHG and ECFHPS strains upon the addition of 10 μg/mL of ampicillin. The left *y*-axis represents OD_600_ of cultures (black circles). Estimated OD_600_ of the VDHG (purple circles) and ECFHPS (green circles) strains was also provided. The left *y*-offset, right *y*-axis and right *y*-offset represent alginate (blue diamond, g/L), 3-HP (red triangles, mg/L) and acetate (grey inverted triangles, g/L) levels, respectively. Source data are provided as a Source Data file.


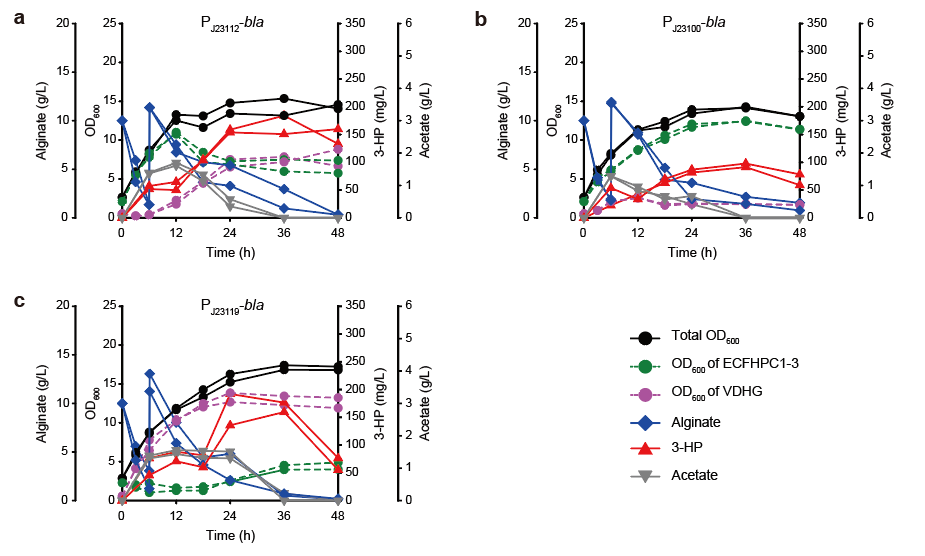


## Supplementary Fig. 8 Fermentation profile of the microbial consortium comprising VDHG and ECFHPC1-3 strains upon the addition of 10 μg/mL of ampicillin.

**(a-d)** The fermentation profile of the co-culture of the VDHG and **(a)** ECFHPC1, **(b)** ECFHPC2, or **(c)** ECFHPC3 strains upon the addition of 10 μg/mL of ampicillin. The left *y*-axis represents OD_600_ of cultures (black circles). Estimated OD_600_ of the VDHG (purple circles) and ECFHPS (green circles) strains was also provided. The left *y*-offset, right *y*-axis and right *y*-offset represent alginate (blue diamond, g/L), 3-HP (red triangles, mg/L) and acetate (grey inverted triangles, g/L) levels, respectively. When the lower-strength promoters (P_J23112_, P_J23100_) were utilized, the ratio of *E. coli* strain was detected at a relatively high level, probably owing to cell lysis of VDHG during a measurement. Source data are provided as a Source Data file.


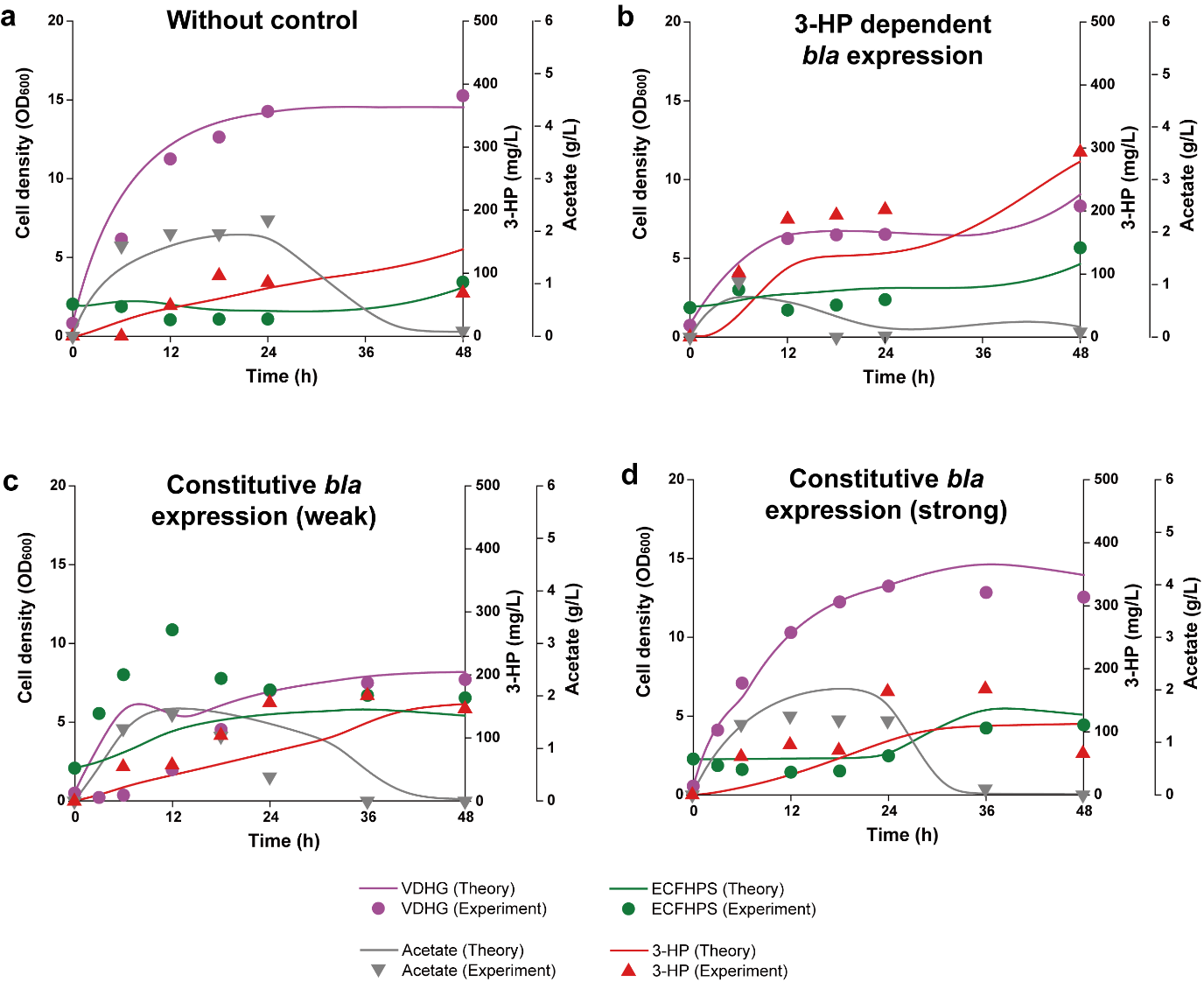


## Supplementary Fig. 9 Modeling results for the co-cultures.

**(a-d)** The simulated profile of cell growth, and production of acetate and 3-HP in three cases: **(a)** without control, **(b)** 3-HP dependent *bla* expression, (**c**) constitutive *bla* expression (weak) (**d**) constitutive *bla* expression (strong). Ampicillin 10 μg/mL was added only in (**b-d**). The *x*-axis represents time (h). The left *y*-axis represents OD_600_ (black circles). The right *y*-axis and *y*-offset represent 3-HP (red triangles, mg/L) and acetate (grey inverted triangles, g/L) levels, respectively. Lines indicate predicted values whereas symbols indicate measured experimental values.

**
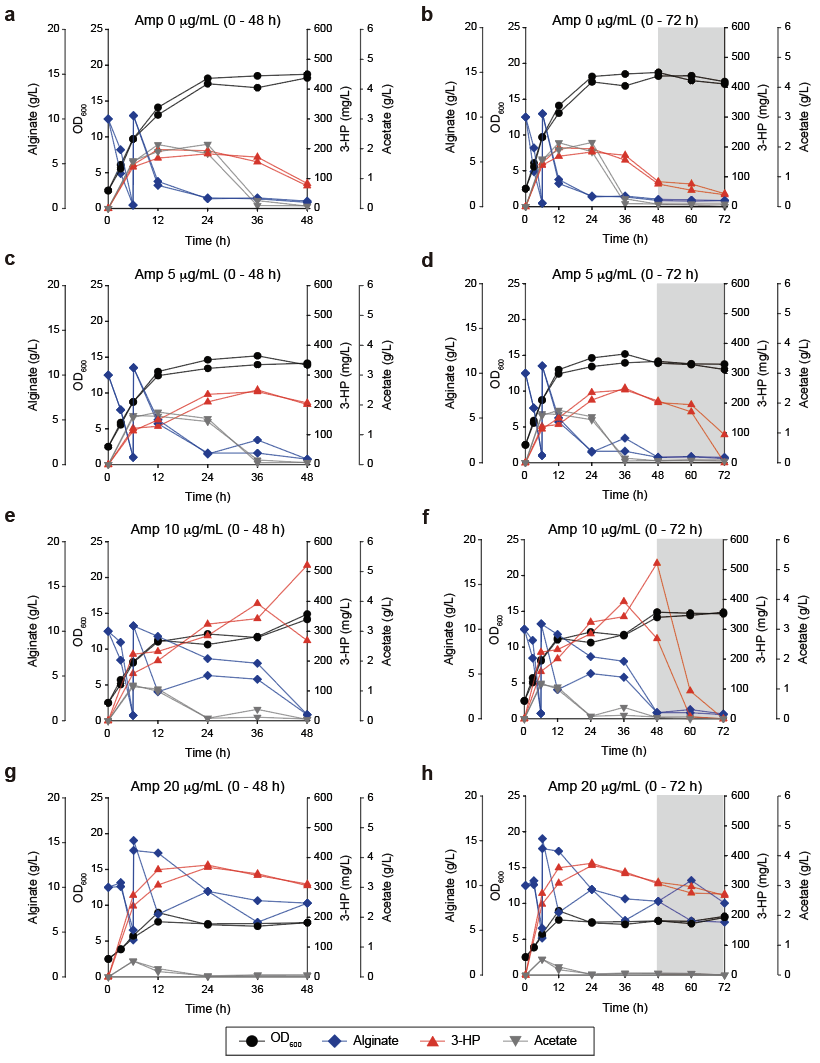
**

## Supplementary Fig. 10. Independent replicative cultures with the population control

Fermentation profiles of the co-culture of the VDHG and ECFHPG strains with the addition of (**a** and **b**) 0, (**c** and **d**) 5, (**e** and **f**) 10, and (**g** and **h**) 20 µg/mL ampicillin during 48 or 72 h, respectively. The left y-axis represents OD_600_ of cultures (black circles). The left y-offset, right y-axis and right y-offset represent alginate (blue diamonds, g/L), 3-HP (red triangles, mg/L) and acetate (grey inverted triangles, g/L) levels, respectively. This data was from independent replicative cultures in the same condition that was used in Figure 4. Source data are provided as a Source Data file.

**Supplementary References**

[1. Rathnasingh, C. *et al.* Production of 3-hydroxypropionic acid via malonyl-CoA pathway using recombinant *Escherichia coli* strains. *J. Biotechnol.* **157**, 633–640 (2012).](https://sciwheel.com/work/bibliography/3263058)

[2. Liu, C., Wang, Q., Xian, M., Ding, Y. & Zhao, G. Dissection of malonyl-coenzyme A reductase of *Chloroflexus aurantiacus* results in enzyme activity improvement. *PLoS ONE* **8**, e75554 (2013).](https://sciwheel.com/work/bibliography/5053098)

[3. Lee, J. H. *et al.* Efficient conversion of acetate to 3-hydroxypropionic acid by engineered *Escherichia coli*. *Catalysts* **8**, 525 (2018).](https://sciwheel.com/work/bibliography/6018283)

[4. Regoes, R. R. *et al.* Pharmacodynamic functions: a multiparameter approach to the design of antibiotic treatment regimens. *Antimicrob. Agents Chemother.* **48**, 3670–3676 (2004).](https://sciwheel.com/work/bibliography/171836)

[5. Smith, H. L. *et al.* Early stages of oxidative stress-induced membrane permeabilization: a neutron reflectometry study. *J. Am. Chem. Soc.* **131**, 3631–3638 (2009).](https://sciwheel.com/work/bibliography/11339412)

[6. Kong, W., Meldgin, D. R., Collins, J. J. & Lu, T. Designing microbial consortia with defined social interactions. *Nat. Chem. Biol.* **14**, 821–829 (2018).](https://sciwheel.com/work/bibliography/5499388)

[7. Seok, J. Y. *et al.* Directed evolution of the 3-hydroxypropionic acid production pathway by engineering aldehyde dehydrogenase using a synthetic selection device. *Metab. Eng.* **47**, 113–120 (2018).](https://sciwheel.com/work/bibliography/5200290)

[8. Seo, S. W. *et al.* Predictive design of mRNA translation initiation region to control prokaryotic translation efficiency. *Metab. Eng.* **15**, 67–74 (2013).](https://sciwheel.com/work/bibliography/1239735)

[9. Woo, S. *et al.* A Vibrio-based microbial platform for accelerated lignocellulosic sugar conversion. *Biotechnol. Biofuels Bioprod.* **15**, 58 (2022).](https://sciwheel.com/work/bibliography/13419155)

[10. Liu, C. *et al.* Functional balance between enzymes in malonyl-CoA pathway for 3-hydroxypropionate biosynthesis. *Metab. Eng.* **34**, 104–111 (2016).](https://sciwheel.com/work/bibliography/5053101)
