# Supplementary material for: Circuit-guided population acclimation of a synthetic microbial consortium for improved biochemical production
Source: Nat Commun. 2022 Nov 7;13:6506. doi: 10.1038/s41467-022-34190-z (PMC9640620; doi:10.1038/s41467-022-34190-z)
Supplement: Supplementary file 2 — Description of Additional Supplementary Files [file 41467_2022_34190_MOESM2_ESM.pdf]

Description of Additional Supplementary Files

File Name: Supplementary Data 1

Description: List of strains and plasmids used in this study

File Name: Supplementary Data 2

Description: List of reagent information and primers used in this study
