# Supplementary material for: Circuit-guided population acclimation of a synthetic microbial consortium for improved biochemical production
Source: Nat Commun. 2022 Nov 7;13:6506. doi: 10.1038/s41467-022-34190-z (PMC9640620; doi:10.1038/s41467-022-34190-z)
Supplement: Supplementary file 3 — Reporting Summary [file 41467_2022_34190_MOESM3_ESM.pdf]

nature portfolio

Corresponding author(s): Gyoo Yeol Jung

Last updated by author(s): Oct 12, 2022

Reporting Summary

Nature Portfolio wishes to improve the reproducibility of the work that we publish. This form provides structure for consistency and transparency in reporting. For further information on Nature Portfolio policies, see our Editorial Policies and the Editorial Policy Checklist.

Statistics

For all statistical analyses, confirm that the following items are present in the figure legend, table legend, main text, or Methods section.

n/a	Confirmed	
☐	☒	The exact sample size (*n*) for each experimental group/condition, given as a discrete number and unit of measurement
☐	☒	A statement on whether measurements were taken from distinct samples or whether the same sample was measured repeatedly
☐	☒	The statistical test(s) used AND whether they are one- or two-sided<br>*Only common tests should be described solely by name; describe more complex techniques in the Methods section.*
☒	☐	A description of all covariates tested
☒	☐	A description of any assumptions or corrections, such as tests of normality and adjustment for multiple comparisons
☐	☒	A full description of the statistical parameters including central tendency (e.g. means) or other basic estimates (e.g. regression coefficient) AND variation (e.g. standard deviation) or associated estimates of uncertainty (e.g. confidence intervals)
☐	☒	For null hypothesis testing, the test statistic (e.g. *F*, *t*, *r*) with confidence intervals, effect sizes, degrees of freedom and *P* value noted<br>*Give P values as exact values whenever suitable.*
☒	☐	For Bayesian analysis, information on the choice of priors and Markov chain Monte Carlo settings
☒	☐	For hierarchical and complex designs, identification of the appropriate level for tests and full reporting of outcomes
☒	☐	Estimates of effect sizes (e.g. Cohen's *d*, Pearson's *r*), indicating how they were calculated

Our web collection on statistics for biologists contains articles on many of the points above.

Software and code

Policy information about availability of computer code

Data collection	Flow cytometry data were collected via CytExpert (version 2.4.0.28, Beckman Coulter).
Data analysis	For modeling, model parameters were obtained using Mathematica. Differential equations were solved using Mathematica's ParametricNDsolve routine (version 12) and experimental data fitting was assisted using Findfit routine (version 12). The computational code are accessible at https://doi.org/10.5281/zenodo.7187480. Statistical analysis were done via SigmaPlot (12.0.Ink, Systat Software). The 5'-UTR sequences were designed using UTR Designer (http://sbi.postech.ac.kr/rbs).

For manuscripts utilizing custom algorithms or software that are central to the research but not yet described in published literature, software must be made available to editors and reviewers. We strongly encourage code deposition in a community repository (e.g. GitHub). See the Nature Portfolio guidelines for submitting code & software for further information.

Data

Policy information about availability of data

All manuscripts must include a data availability statement. This statement should provide the following information, where applicable:

- Accession codes, unique identifiers, or web links for publicly available datasets
- A description of any restrictions on data availability
- For clinical datasets or third party data, please ensure that the statement adheres to our policy

A reporting summary for this Article is available as a Supplementary Information file. The strains and plasmids used in this study are provided as a Supplementary. Synthetic promoter sequences were obtained from Registry of Standard Biological Parts (http://parts.igem.org/Main_Page).

Human research participants

Policy information about studies involving human research participants and Sex and Gender in Research.

Reporting on sex and gender	N/A
Population characteristics	N/A
Recruitment	N/A
Ethics oversight	N/A

Note that full information on the approval of the study protocol must also be provided in the manuscript.

Field-specific reporting

Please select the one below that is the best fit for your research. If you are not sure, read the appropriate sections before making your selection.

☒ Life sciences ☐ Behavioural & social sciences ☐ Ecological, evolutionary & environmental sciences

For a reference copy of the document with all sections, see nature.com/documents/nr-reporting-summary-flat.pdf

Life sciences study design

All studies must disclose on these points even when the disclosure is negative.

Sample size	Sample sizes were determined to be either 2 or 3 depending on experimental availability at a given time. Reproducibility was confirmed by repeated cultures. Growth control test using population guider was from three biological replicates (Fig. 3). Fermentation data of microbial consortia was from two biological replicates (Fig. 2 and Fig. 4a-h). The data of Supplementary Fig. 10 are independent replicative cultures in the same condition that was used in Fig. 4. The sample size was provided in figure legends.
Data exclusions	No data was excluded from the analysis except culture samples collected at 36 h in Figure 2 and 4. We provided results of independent cultures in Figure S10.
Replication	All experiments shown in the same figure was performed side by side at the same time. In the case of Fig. 4, independent replicative cultures were performed, and related data are provided in Supplementary Fig. 10.
Randomization	Replicates of the different strains were treated and grown at the same time without any randomization. We compared each fermentation parameters respective to the untreated culture sample.
Blinding	C.W.K. collected data for most experiments and all other investigators were participated with blinding during data collection.<br>For analysis, blinding was not necessary because the sample number was small and differences were clear between strains.

Reporting for specific materials, systems and methods

We require information from authors about some types of materials, experimental systems and methods used in many studies. Here, indicate whether each material, system or method listed is relevant to your study. If you are not sure if a list item applies to your research, read the appropriate section before selecting a response.

Materials & experimental systems

n/a	Involved in the study
☒	☐ Antibodies
☒	☐ Eukaryotic cell lines
☒	☐ Palaeontology and archaeology
☒	☐ Animals and other organisms
☒	☐ Clinical data
☒	☐ Dual use research of concern

Methods

n/a	Involved in the study
☒	☐ ChIP-seq
☐	☒ Flow cytometry
☒	☐ MRI-based neuroimaging

Flow Cytometry

Plots

Confirm that:

☒ The axis labels state the marker and fluorochrome used (e.g. CD4-FITC).

☒ The axis scales are clearly visible. Include numbers along axes only for bottom left plot of group (a 'group' is an analysis of identical markers).

☒ All plots are contour plots with outliers or pseudocolor plots.

☒ A numerical value for number of cells or percentage (with statistics) is provided.

Methodology

Sample preparation	Bacterial cultures were washed using fresh minimal medium to obtain OD600 value of 1. A Beckman Coulter flow cytometer was used to measure mCherry and sGFP signal. For each sample, the fluorescence signals of at least 100,000 cells were monitored.
Instrument	Beckman Coulter flow cytometer (Brea, CA, United States).
Software	CytExpert version 2.3.
Cell population abundance	The samples were not sorted.
Gating strategy	Cells were gated in the FSC vs. SSC plot using a tetragonal gate to analyze major populations.

☒ Tick this box to confirm that a figure exemplifying the gating strategy is provided in the Supplementary Information.
